# Role of the microbiome in occurrence, development and treatment of pancreatic cancer

**DOI:** 10.1186/s12943-019-1103-2

**Published:** 2019-12-01

**Authors:** Yicheng Wang, Gang Yang, Lei You, Jinshou Yang, Mengyu Feng, Jiangdong Qiu, Fangyu Zhao, Yueze Liu, Zhe Cao, Lianfang Zheng, Taiping Zhang, Yupei Zhao

**Affiliations:** 1 Department of General Surgery, Peking Union Medical College Hospital, Chinese Academy of Medical Sciences and Peking Union Medical College, No. 1 Shuaifuyuan, Wangfujing Street, Beijing, 100730 China; 2 Department of Nuclear Medicine, Peking Union Medical College Hospital, Chinese Academy of Medical Sciences and Peking Union Medical College, Beijing, 100730 China; 3 Clinical Immunology Center, Chinese Academy of Medical Sciences and Peking Union Medical College, Beijing, 100730 China

**Keywords:** Pancreatic cancer, Microbiomes, Chemotherapy, Diagnosis

## Abstract

Pancreatic cancer is one of the most lethal malignancies. Recent studies indicated that development of pancreatic cancer may be intimately connected with the microbiome. In this review, we discuss the mechanisms through which microbiomes affect the development of pancreatic cancer, including inflammation and immunomodulation. Potential therapeutic and diagnostic applications of microbiomes are also discussed. For example, microbiomes may serve as diagnostic markers for pancreatic cancer, and may also play an important role in determining the efficacies of treatments such as chemo- and immunotherapies. Future studies will provide additional insights into the various roles of microbiomes in pancreatic cancer.

## Background

**Fig. 1:**
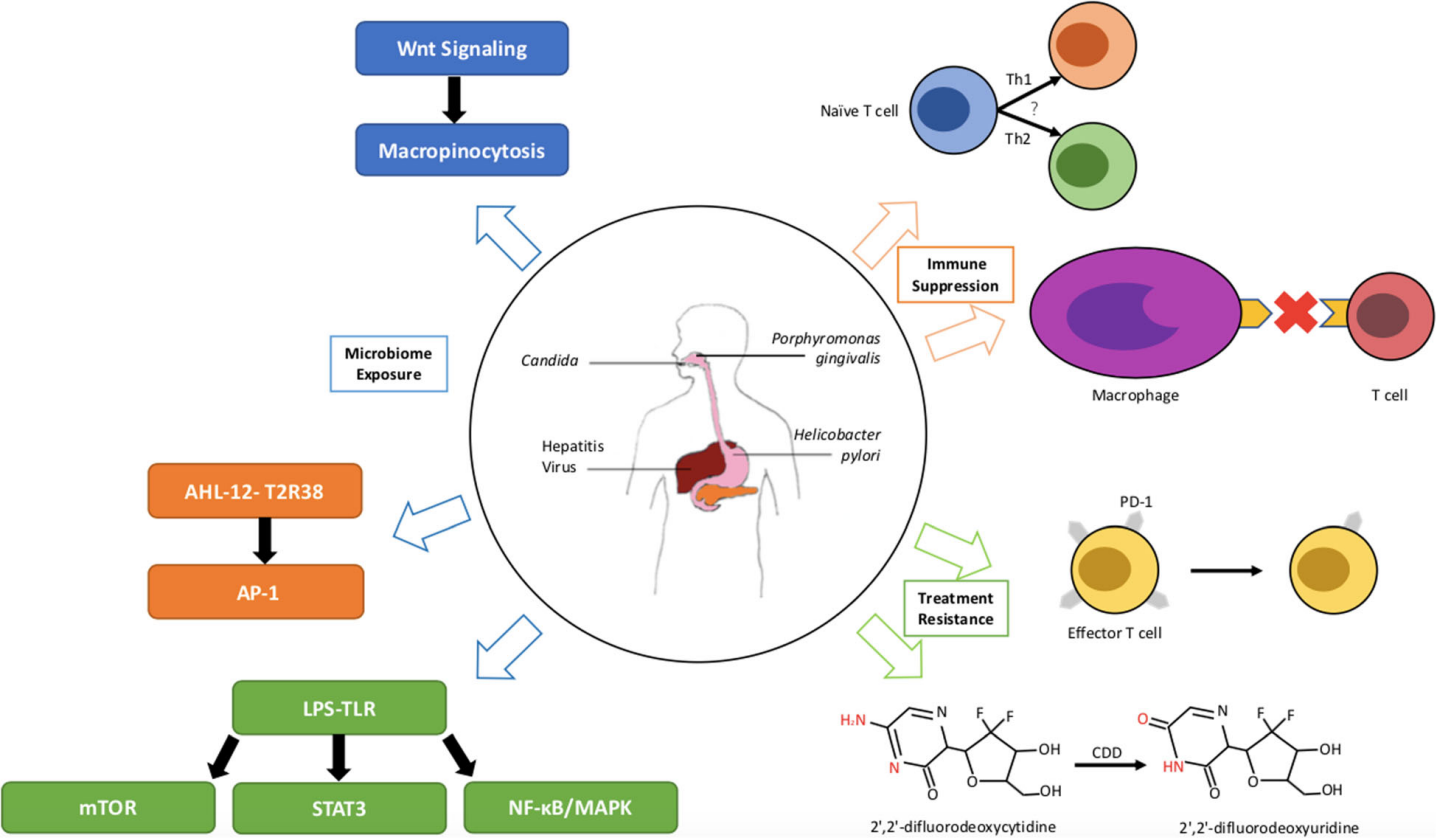
Microbiomes play important roles in the development and treatment of pancreatic cancer. The blue arrow indicates that microbiome exposure activates inflammation to promote development of pancreatic cancer. The orange arrow shows that the microbiome leads to immune suppression. The green arrow shows that the microbiome influences the effects of cancer treatments. AHL-12, N-acetyl-dodecanoyl homoserine; T2R38, one of the family of bitter receptors; mTOR, mammalian target of rapamycin; LPS, lipopolysaccharide; TLR, Toll-like receptor; AP-1, Activator protein 1; STAT3, Signal transducers and activators of transcription 3; Th1/2, helper T cell 1/2; PD-1, programmed cell death-1; CDD, cytidine deaminase; 2′,2′-difluorodeoxycytidine, gemcitabine; 2′,2′-difluorodeoxyuridine, an inactive form of gemcitabine

Pancreatic cancer (PC), one of the most lethal malignancies, is the 10th most frequent cancer in men and the 9th most common in women in the United States. PC is responsible for the third-highest number of cancer-related deaths [1]. The incidence of PC has shown a rapid upward trend in recent years. PC onset is difficult to detect, and early symptoms are atypical. Many patients are diagnosed with local progression or distal metastasis and are not candidates for surgery, leading to a 5-year survival rate of less than 9% [1]. Therefore, it is very important to better understand the occurrence and development of PC to enable early diagnosis and treatment. In recent years, associations between microbiomes and the occurrence and development of PC have been identified, potentially representing an early screening and risk assessment factor. Furthermore, inflammation and immunosuppression caused by microbiome changes are recognized as mechanisms associated with cancer development [2–4]. In addition, the microbiome may also affect the metabolism of chemotherapy drugs, thereby modulating the effects of chemotherapy [4, 5]. This review will summarize these complex issues (Fig. 1).

## Roles of microbiomes in development of PC

Infectious factors play a causative role in approximately 10–20% of all cancers worldwide [6]. However, in PC, no microbe has been identified as a causative agent. Many studies have suggested that changes in the diversity, proportions and dominant organisms of the microbiome (*Porphyromonas*, *Actinomycetes*, *Neisseria*, *Streptococcus*, *Bacteroides Bifidobacteria* and *Fusobacterium* species) may be associated with the occurrence and development of PC [7–14]. Particularly, Riquelme et al. used 16S rRNA gene sequencing to analyze the tumor microbiome composition of PDAC patients with short-term survival and long-term survival [15]. They supported *Pseudoxanthomonas-Streptomyces-Saccharopolyspora-Bacillus clausii* can highly predict the long-term survivorship, which showed the effect of microbiome on prognosis. Although it remains unclear whether these microbiome properties are directly associated with PC, the studies summarized in Table 1 have demonstrated some preliminary correlations.

**Table 1:** Human studies investigating the role of microbiomes in pancreatic cancer

Study design	Patients or Samples	Content	Conclusion	Refs
Case-control study	·HOMIM: 10 PC & 10 controls	·16 of 410 bacterial taxa	Significant changes observed in the microbial composition between pancreatic cancer patients and healthy controls.	[11]
	·qPCR: 28 PC & 27 chronic pancreatitis patients & 28 controls	·*Neisseria elongata* and *Streptococcus mitis*		
Meta-analysis	8 studies of periodontitis or edentulism	RR for periodontitis and PC was 1.74 (95% CI 1.41–2.15] and 1.54 (95% CI 1.16–2.05) for edentulism	Both periodontitis and edentulism appear to be associated with PC, even after adjusting for common risk factors.	[16]
Prospective cohort study	Blood samples from 405 PC & 416 controls	Antibodies against *Porphyromonas gingivalis* ATCC 53978	Individuals with high levels of antibodies against *Porphyromonas gingivalis* ATCC 53978 had higher risk of PC.	[17]
Case-control study	16S rRNA of 30 PHC patients and 25 healthy controls	Microbiome diversity of the tongue coat	The microbiota dysbiosis of the tongue coat in PHC patients was identified.	[18]
Cohort study	Cyst fluid and plasma of suspected PCN	Bacterial 16S DNA copy number and IL-1β	Intracystic bacterial 16S DNA copy number and IL-1β protein quantity were significantly higher in IPMN.	[19]
Case-control study	Blood samples from 92 PC & 30 gastric cancer & 35 colorectal cancer & 27 controls	IgG antibodies against *Hp*	Suggested an association between *Hp* infection and pancreatic cancer.	[20]
Case-control study	·16S rRNA gene of 14 PC & 14 controls	*Hp*, IL-6 and CRP	PC patients had higher IL-6 and CRP in blood and a higher incidence of *Hp* in duodenum	[21]
	·Blood samples			
	·Urea breath test			
Meta-analysis	Blood samples of 580 PC & 626 controls	*Hp* and CagA	The evidence of CagA strain-specific associations is respective.	[22]
Meta-analysis	117 meta-analytical or pooled reports of the association between specific risk factors and PC risk.	*Hp* has estimated population attributable fractions is 4–25%.	*Hp* infection is the major risk factors associated with PC.	[23]
Meta-analysis	1003 PC & 1754 controls in 8 case-control studies	OR = 1.45 (95% CI: 1.09–1.92) between *Hp* and PC under the random effects model.	*Hp* infection can significantly increase the risk of developing pancreatic cancer.	[24]
Meta-analysis	2335 patients in 6 studies	AOR = 1.38 (95%CI: 1.08–1.75; *P* = 0.009) between *Hp* and PC	A significant association between *Hp* seropositivity and development of pancreatic cancer was seen	[25]
Meta-analysis	1083 PC & 1950 controls in 9 studies	OR = 1.47 (95%CI: 1.22–1.77) between *Hp* and PC	*H. pylori* infection is significantly, albeit weakly, associated with pancreatic cancer development.	[26]
Nested case-control study	104 cases randomly selected subjects among 507 developed PC, 262 cases from 730 controls	*Hp* and its CagA protein	*Helicobacter pylori* infection is not associated with development of PC.	[27]
Prospective cohort study	87 PC & 263 controls from residents born from 1921 to 1949 in Malmö, Sweden	*Hp*	No association between *Hp* infection and the risk for PC was found.	[28]
Meta-analysis	65,155 observations in 3 cohort studies and 6 nested case-control studies	OR = 1.09(95%CI: 0.81–1.47)	The linkage of PC to *Hp* infection was not warranted on the whole.	[29]
Prospective cohort study	19,924 participants including 126 PC	*Candida*	Individuals with Candida-related lesions had a 70 80% excess risk of developing PC.	[30]
Population-based cohort study	34,829 patients from the National Health Insurance system of Taiwan	*Candida*	The risks of pancreatic cancer was significantly higher in the *Candida* Infection group.	[31]

*AOR*, adjusted odds ratio; *CagA*, cytotoxin-associated gene-A; *CI*, confidence interval; *CRP*, C-reactive protein; *HOMIM*, Human Oral Microbiological Identification Microarrays; *Hp,* Helicobacter pylori; *IgG*, Immunoglobulin G; *IL*, interleukin; *IPMN*, intraductal papillary mucinous neoplasm; *PC*, pancreatic cancer; *PCN*, pancreatic cystic neoplasm; *PHC*, pancreatic head carcinoma; *Porphyromonas gingivalis* ATTC 53978, a pathogenic periodontal bacteria; qPCR, Real-time quantitative polymerase chain reaction; *RR*, relative risk

### The oral microbiome and PC

The oral cavity is a large reservoir of microbes including more than 700 types of bacteria, viruses and fungi [16, 32]. When conditions change, the commensal microbiomes can become pathogenic and lead diseases including PC [16]. Periodontal disease, an inflammation caused by oral microbes, has been regarded as a risk factor for PC. For example, Maisonneuve et al. conducted a meta-analysis of eight studies and suggested a significant link between periodontal disease and increased risk of PC [33]. The relative risks for PC in individuals with periodontitis and edentulism were 1.74 (95% confidence interval, CI 1.41–2.15) and 1.54 (95%CI 1.16–2.05), respectively [33]. Farrell et al. conducted an analysis using human oral microbiological microarrays to study variation in salivary microbiomes among 10 patients with resectable PC and 10 matched healthy controls. They identified 410 bacterial taxa [11], including 16 (3.9%) organisms such as *Neisseria elongata* and *Streptococcus mitis* whose frequencies differed significantly between the two groups. This study revealed significant changes in the microbial composition of PC patients and healthy controls [11]. In addition, *Porphyromonas gingivalis*, a commonly identified bacterium in patients with periodontal disease, is thought to increase risk of developing PC [17, 34]. To understand the connection between oral microbiomes and PC, Michaud et al. measured antibodies against oral bacteria in pre-diagnostic blood samples from 405 PC patients and 416 matched controls nested in the European Prospective Investigation into Cancer and Nutrition (EPIC) study [35]. The results showed that individuals with high levels of antibodies against *P. gingivalis* ATCC 53978 were at two-fold increased risk of PC compared with individuals with lower levels of these antibodies [35]. In addition, the authors found that individuals with consistently high levels of antibodies to common oral bacteria were at 45% lower risk for PC compared to those with lower antibody levels [35]. Similarly, other studies using 16S rRNA sequencing suggested that the presence of *Haemophilus, Porphyromonas, Leptotrichia* and *Fusobacterium* species in the oral cavity was also associated with increased risk of PC in humans [9, 18, 36].

As one of star oral microbiomes associated with PC, *P. gingivalis* has been extensively studied. Although several studies have shown that *P. gingivalis* is a new risk factor for PC, further researches are needed to explore the specific mechanisms leading to PC. Some researchers hypothesized that one potential mechanism might be that a peptidyl-arginine deiminase enzyme secreted by *P. gingivalis* leads to p53 and K-ras mutations following degradation of arginine [19], while others focus on the effect of oral bacteria on the systemic immune response, including IL-1β, IFNγ, and TNF [2, 37]. How, then, can the oral microbiome affect the pancreas? Gaiser et al. found higher loads of oral bacterial DNA in the cyst fluid of intraductal papillary mucinous neoplasms (a condition which can progress to PC), providing support for the relationship between oral and pancreatic microbes [20]. By feeding wild-type mice fluorescently-labeled *Enterococcus faecalis* or *Escherichia coli*, Pushalkar et al. found these bacteria accrued in the pancreas of mice, demonstrating that microbes can migrate to the pancreas and directly affect the pancreatic microenvironment [4]. However, no clear evidence has indicated which channels microbes use to reach the pancreas. In fact, although the pancreas belongs to distal organ of the digestive tract, it is still connected to the digestive tract via duodenum. Therefore, oral microbes are likely to enter the pancreas through the digestive tract. In addition, it is well known that oral microorganisms easily enter the blood, leading to bacteremia. Therefore, oral microorganisms may also enter the pancreas through blood circulation. In summary, the exact channel of oral microbes into the pancreas requires further researches to determine.

### Helicobacter pylori and PC

*Helicobacter pylori*, a well-known bacterium that colonizes the human stomach, has been the subject of increasing attention over the last 30 years [38]. Many previous studies have attempted to correlate the presence of *H. pylori* with PC using serologic and culture-based methods. However, the vast majority of commensal microbes cannot be cultured, affecting the objective investigation of their role in pancreatic diseases [21, 39–42]. New techniques, such as next-generation sequencing and metagenomics, have provided a more representative assessment of the microbial community in health and disease and the dynamic interactions between microbiomes and their human hosts [22]. These techniques may help in understanding the association between *H. pylori* and PC. To determine whether *H. pylori* infection was associated with PC, Raderer et al. used enzyme-linked immunosorbent assays to analyze IgG antibodies against *H. pylori* in blood samples from 92 patients with PC, 30 patients with gastric cancer, 35 patients with colorectal cancer, and 27 healthy volunteers [39]. The results showed that 65% of patients with PC and 69% of patients with gastric cancer were seropositive, compared with only 45% of the other individuals [39]. Mei et al. found that *H. pylori* could be detected in the duodenum at higher frequencies in PC patients than in healthy controls [23]. Similarly, other studies used meta-analysis to confirm that *H. pylori* was associated with increased risk of PC in humans [24–26, 43, 44]. The probable mechanism of microbiome transmission involves changes in the intestinal microbial environment, which can alter the composition of the intestinal microbiome, increase intestinal permeability and permit microbial access to the bloodstream and distant organs [27, 45]. *H. pylori* may promote the development of PC by causing chronic mucosal inflammation as well as changes in cell proliferation and differentiation [28].

However, several other studies concluded that *H. pylori* was not associated with PC [25, 29, 46, 47]. One of the potential explanations for this discrepancy is that *H. pylori* has several variants, of which cytotoxin-associated gene A (Cag-A) positive strains have been the best studied. Cag-A has been linked to multiple diseases such as gastric inflammation and ulceration, gastric cancer and PC [48, 49]. However, it remains controversial whether Cag-A-positive or Cag-A-negative strains are associated with PC [50, 51]. An effect modification by ABO blood type was reported in a large case-control study: the association between PC and Cag-A-negative *H. pylori* was evident only in individuals with non-O blood types [41]. This finding may be explained by differences in the terminal antigens of gastrointestinal mucins in individuals with non-O blood types, which affects binding by *H. pylori* [2].

No single clear mechanism has been widely accepted to explain associations between *H. pylori* and PC. One hypothesis suggests that *H. pylori* infection leads to hyperchlorhydria and enhanced release of secretin, promoting pancreatic hyperplasia [52]. Conversely, another hypothesis suggested that *H. pylori* infection led to atrophic gastritis and hypochlorhydria, resulting in bacterial overgrowth and overproduction of N-nitrosamines [53]. In summary, the role of *H. pylori* in PC remains unclear. Further studies are required to consider other potential confounding risk factors and conclusively explore whether *H. pylori* is truly associated with the occurrence and development of PC.

### The pancreatic microbiome

Traditionally, the pancreas has not been considered to have its own microbiome. Li et al. performed bacterial 16S rRNA gene-specific PCR to analyze the microbial constituents in the pancreatic cyst fluids, where *Bacteroides*, *Escherichia/Shigella*, and *Acidaminococcus* were predominant [30]. They reflected the local microbiota in the pancreas, and prove that pancreatic cyst fluid is a very important sample for microbial identification. Now, in addition to *P. gingivalis* and *H. pylori*, other microbes have been identified in PC tissues. Using 16S rRNA gene sequencing, Pushalkar et al. found high proportions of *Proteobacteria* (45%), *Bacteroidetes* (31%), and *Firmicutes* (22%) species in PC tissues [4]. Interestingly, they concluded that the microbiome proportions in PC tissue were quite different from those of normal pancreatic tissue. Some recent research also showed similar results [31, 54]. Thus, the pancreas is not sterile and has its own microbial environment which may affect the occurrence and development of PC. More complex mechanisms involving a large number of factors may alter the pancreatic microbiome. These alterations may occur via natural and non-natural channels.

### Fungi and viruses in PC

Some studies have linked fungi and viruses to the development of PC. For example, a prospective cohort study conducted in Sweden showed that *Candida* infection in the oral cavity was associated with development of PC [55]. Similarly, another population-based cohort study in Taiwan showed that risk of PC was significantly higher among *Candida*-infected individuals [56]. However, the relationships between fungal infections and PC require further study. The roles of hepatitis viruses in the development of hepatocellular carcinoma are relatively clear. However, some evidence suggested that hepatitis viruses may also be associated with PC. Katakura et al. found increased serum levels of pancreatic enzymes in viral hepatitis patients [57], while Jin et al. identified a link between hepatitis B virus and chronic pancreatitis [58]. These studies link chronic hepatitis, chronic pancreatitis and PC and demonstrate that a potential role of viruses in PC cannot be ignored.

## Mechanisms of microbiome involvement in development of PC

### Microbes and pancreatic inflammation

Microbial infections often lead to inflammation [59]. Sometimes inflammation is a protective response to factors such as pro-inflammatory mediators, environmental toxins, or chronic infection [60–62]. However, inflammation is also a risk factor for development of many cancers. For example, Dejea et al. demonstrated that patients with familial adenomatous polyposis had higher frequencies of *E. coli* and *Bacteroides fragilis* cells in the colonic mucosa compared with healthy individuals. Moreover, tumor-prone mice had higher interleukin-17 (IL-17) and IL-23 levels and developed tumors faster upon stimulation by microbes [63, 64]. Inflammation of the pancreas also increases the incidence of PC. Patients with hereditary autoimmune pancreatitis are estimated to carry a lifetime risk of 40% of developing PC and patients with chronic pancreatitis, a recognized risk factor for PC, have a 13-fold higher risk of PC than other individuals [65, 66]. Multiple cohort studies have shown that acute pancreatitis is also associated with the development and progression of PC [67–69]. However, acute pancreatitis is not a direct factor, but the chronic prolongation of inflammation leads to the occurrence and development of PC [68]. Although there are no identified pathogens, this type of chronic inflammation can also be caused by microbial infections [70]. Microbial-induced inflammation leads to tumorigenesis through activation of tumor-related inflammatory signaling pathways.

#### Macropinocytosis and Wnt signaling

Microbes can trigger macropinocytosis, an endocytic process used by cells for antigen capture and presentation, to activate inflammation [71, 72]. For example, Bacillus Calmette-Guérin (BCG) can be internalized through macropinocytosis to treat bladder cancer [73, 74]. Moreover, *Salmonella* species can invade mammalian cells by inducing macropinocytosis through actin remodeling [72, 75]. Importantly, the process of macropinocytosis is closely related to the Wnt (Wingless/Integrated) signaling pathway, which is important for cell proliferation and differentiation during tumorigenesis, including PC [76]. For instance, Redelman-Sidi et al. demonstrated that Wnt-driven macropinocytosis occurred downstream of the β-catenin–dependent canonical Wnt pathway and was PAK1 dependent, contributing to cancer growth during the early phases of oncogenesis [77]. In PC cells, Wnt pathway activation is also relevant to macropinocytosis [71]. Although the details of this mechanism require further study, the influence of the Wnt signaling pathway on microbial uptake is clear.

#### Lipopolysaccharide stimulation of toll-like receptors may link microbiomes to inflammation

Dysbiotic microbial compositions could also interact with some receptors in cells to active inflammation and promote tumorigenesis. Lipopolysaccharide (LPS), a Gram-negative bacterial cell wall component, is specifically recognized by Toll-like receptor 4 (TLR4), a family member of pattern recognition receptors (PRR) [10, 78]. The interaction between LPS and TLR4 can activate the secretion of downstream pro-inflammatory cytokines [78], linking microbes to inflammation. Below, we discuss several molecules related to LPS-TLR signaling and their relevance to PC.

Physiological disorders resulting in destruction of the gut microbiome can lead to inflammatory conditions and some types of cancer. These pathologies are controlled by mammalian target of rapamycin (mTOR) [79], which is a downstream effector of TLRs [80]. Moreover, mTOR also plays a vital role in tumorigenesis, including in PC [81–83]. Phosphorylation of Extracellular regulated protein kinases 1/2 (ERK1/2) and mTOR was inhibited and pancreatic tumor size was reduced in mice if the engrafted pancreatic tumor cells were cultured in engineered-resistant starch [79, 84], which can shape the composition of the gut microbiomes. Therefore, the gut microbiome can influence the mTOR pathway and promote PC.

**Fig. 2:**
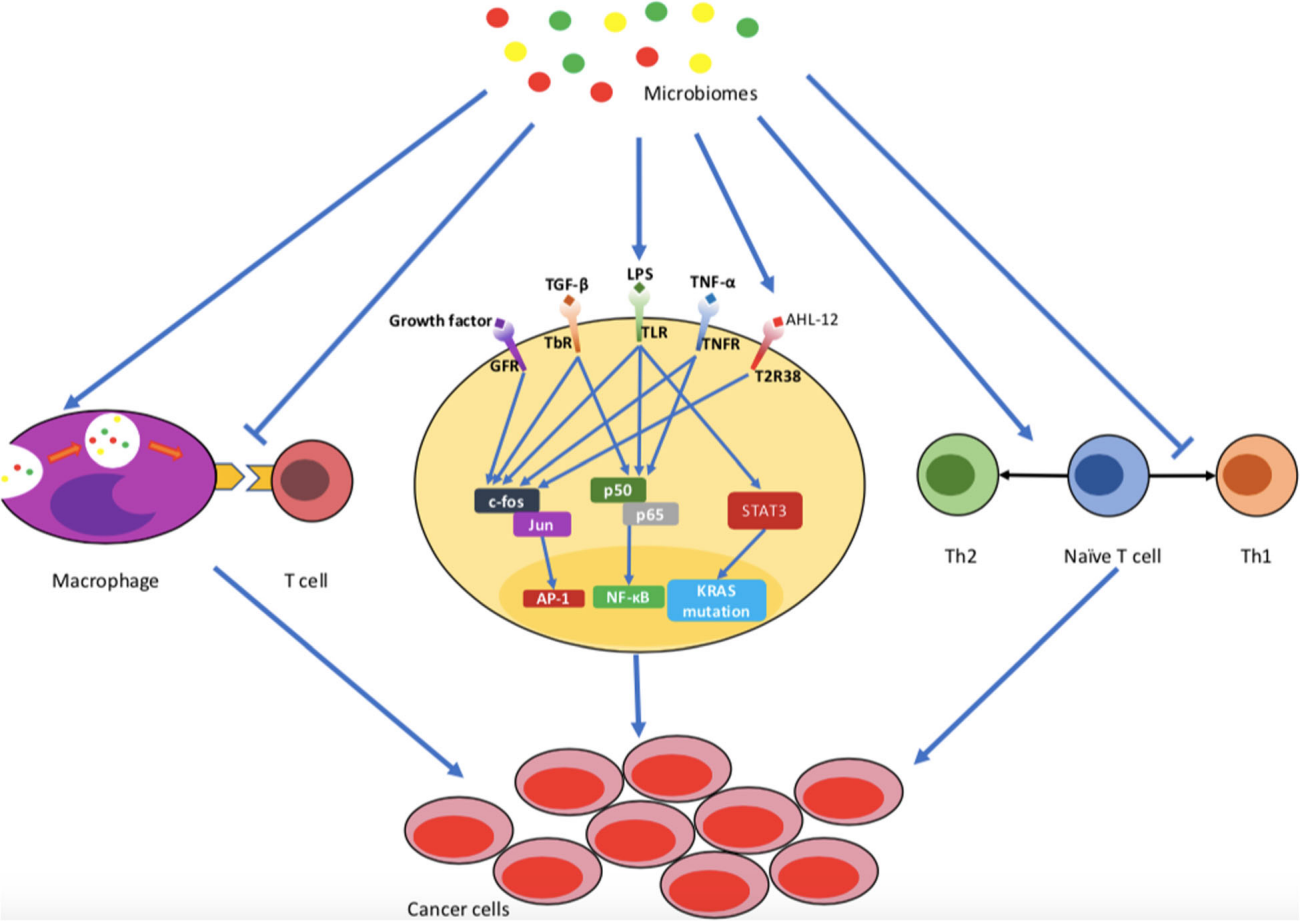
Microbiomes are involved in the occurrence pancreatic cancer. Microbiomes can lead to development of inflammation, inhibit interactions between macrophages and T cells, and favor Th2 polarization of the T cell response. All of these factors can contribute to the occurrence of pancreatic cancer. GFR, growth factor receptor; TGF-β, transforming growth factor-β; TbR, transforming growth factor-β receptor; TLR, Toll-like receptor; TNF-α, tumor necrosis factor-α; TNFR, tumor necrosis factor receptor

The nuclear factor kappa B/mitogen-activated protein kinase (NF-κB/MAPK) signaling pathway plays a major role in inflammation [17]. The NF-κB/MAPK signaling pathway, whose core complexes are c-fos/Jun and p50/p65, is associated with both inflammation and tumorigenesis (Fig. 2). The interaction between LPS and TLR4 can activate both NF-κB and Activator protein 1 (AP-1), leading to expression of pro-inflammatory cytokines and dysregulation of cellular processes [85, 86]. Similar to the role of LPS-TLR signaling in inflammation, Beller et al. found that *Pseudomonas aeruginosa* N-acetyl-dodecanoyl homoserine (AHL-12) could also activate downstream AP-1 by binding to the bitter receptor T2R38, which was initially identified in taste bud cells in the oral cavity [87–89]. Expression of T2R38 has also been described in pancreatic tumor cells [90]. Thus, T2R38 may represent one bridge connecting the microbiome with PC. Further research is needed to explore the relationship between T2R38 and PC.

Other molecules are involved in crosstalk between inflammation and tumorigenesis. For example, LPS-TLR signaling can also activate the STAT3 (Signal transducers and activators of transcription 3) pathway and trigger mutation of the Kirsten rat sarcoma viral oncogene (KRAS), which can promote PC progression [91–93] (Fig. 2). Therefore, these studies have found initial links between microbiomes, inflammation and PC, suggesting that microbial-associated inflammation could play an important role in development of PC.

### Microbiomes and the immune system in PC

The occurrence and development of tumors are closely related to the immune system, and the impact of microbiomes on the immune system is a very hot topic. Recently, Riquelme et al. used human-into-mice fecal microbiota transplantation experiments from short-term survival, long-term survival, or control donors, and they found modulating the tumor microbiome can affect tumor growth as well as tumor immune infiltration [15]. Different studies have highlighted the various roles of microbiomes in the immune system, including effects on immune maturation and immune suppression.

The gut microbiomes and the immune system can affect one another in the gut lamina propria, and similar effects have recently been described at extraintestinal sites [94, 95]. Round et al. found that the immune systems of germ-free mice were deficient, with hypoplastic lymphoid organs and impaired immune cells. However, the immune systems of these mice matured after gut microbiome transplantation from specific-pathogen-free mice [96]. Mechanistically, microbiomes can act as antigens and activate the immune system. Damage to the intestinal mucosa allows microbes to enter the gut lamina propria and migrate to distant lymphoid organs, resulting in activation of the immune system. However, in the absence of intestinal microflora, the immune system cannot be activated [94, 97]. Responses to gut microbiomes mediated by IL22^+^ innate lymphoid cells, Th17 cells and regulatory T cells occurred in mice deficient in adaptive immunity, indicating that the gut microbiome can promote innate immunity [98]. Several studies showed that specific microbes, such *B. fragilis* and *Bifidobacterium* species, may be important factors for maturation of the immune system [99–101]. In lung cancer, bladder cancer, kidney cancer and melanoma, similar results have been reported [102–104].

However, other studies showed the opposite result, finding that immune systems became activated during antibiotic treatment in models of liver cancer, colon cancer and melanoma [105–107]. Immune cells are essential in the PC microenvironment, where they promote tumorigenesis along with related inflammatory factors, and thus immune cell infiltration has further effects on disease progression [95, 108]. Several studies have focused on immune cell infiltration in PC. Infiltration by different leukocyte subsets can have different effects on tumorigenesis and progression, either promoting tumor growth or inhibiting tumor progression [109]. Some studies have shown that Th1-polarized CD4^+^ and CD8^+^ T cells inhibit pancreatic tumor growth in a mouse model and are associated with prolonged survival in human PC [94, 106, 110]. In contrast, antigen-specific Th2-polarized CD4^+^ T cells can promote progression of PC in mice [106, 111], and are associated with shorter survival in human PC [112] (Fig. 2). Another study found that FOXP3^+^ regulatory T cells can promote immune escape in PC [113]. However, differentiation of T cells may be regulated by microbiome composition. For example, the number of pancreas-infiltrating CD45 immune cells was reduced in antibiotic-treated NOD/SCID (Non-obese diabetic/severe combined immunodeficient) mice [107]. In KC mice (K-ras^LSL.G12D^; Pdx1-Cre mice) and KPC mice (K-ras^LSL.G12D^; p53R^172H/+^; Pdx1-Cre mice), myeloid-derived suppressor cell infiltration was reduced during antibiotic treatment. Moreover, Th1 polarization of CD4^+^ T cells and cytotoxicity of CD8^+^ T cells were enhanced as shown by high T-BET, tumor necrosis factor (TNF)-α and interferon-γ expression [4] (Fig. 2).

Microbe-mediated immune suppression was associated with pattern recognition receptors, and inhibition of these receptors slowed tumor development [114]. As mentioned above, following interaction with LPS, TLR4 can activate the downstream NK-κB/MAPK pathway and lead to development of PC (Fig. 2). Several TLRs (TLR2, TLR4, TLR5 and TLR7) are associated with suppression of innate and adaptive immunity to promote development of PC [4, 111, 115]. Mechanistically, activation of TLRs results in inhibition of interactions between macrophages and lymphocytes, which are abrogated in the absence of TLRs signaling [4].

These opposite results may indicate that different microbiomes play different roles in immunity or in different tumor models. It is clear that further studies are needed to target the PC-associated microbiome to enhance immunotherapy. Moreover, the composition of the gut microbiome can divide patients into responders and nonresponders for immunotherapy, which demonstrates the significance of exploring specific microbial features as diagnostic markers in PC.

### Microbiomes and metabolism in PC

Microbiomes play an important role in the body, especially in the metabolism of sugars, amino acids and fats as well as synthesis of vitamins or other nutrients [116, 117]. Microbiomes cause changes in human metabolism, contributing to a variety of metabolic diseases such as obesity and diabetes. Obesity and diabetes are also important factors in the development of PC. Papamichael et al. reported that colonization by *H. pylori*, which is a potential independent risk factor for PC, may be associated with obesity and diabetes [118]. Therefore, microbiomes also affect the development of PC via changes in metabolism.

Obesity is a risk factor for PC in both men and women [119–122]. Obesity affects the progression of pancreatic tumors by modifying the interactions between adipocytokines [123, 124], adiponectin [125–127], deoxycholic acid [128], and many other molecules. Furthermore, the gut microbiome is also believed to play an important role in connecting obesity and PC. Donohoe et al. found that the body mass indices of lean mice could be increased by transplantation of gut microbiomes from obese animals, which were able to digest more nutrients [129]. Therefore, we speculate that microbiomes may participate in the occurrence and development of cancer through some metabolic mechanisms. In addition to changes in microbial diversity [130], some microbial metabolites may be associated with the development of obesity. For example, short-chain fatty acids (SCFAs), which are enriched in obese individual, can activate the MAPK signaling pathway through G-protein-coupled receptors and lead to cancer cell proliferation [86]. In addition, obesity can promote release of LPS from the gut microbiome and therefore lead to endotoxemia [131]. Ren et al. demonstrated that PC patients had more LPS-producing bacteria than healthy controls, supporting a potential relationship between endotoxemia and PC [8]. In fact, obesity is also a type of inflammatory state. As mentioned above, microbiomes can influence the development and progression of PC through different mechanisms, and the NF-κB pathway is a common pathway in both inflammation and cancer. Pagliari et al. suggested that obesity is associated with the release of various pro-inflammatory cytokines, such as IL-6 and TNF, which activate the NF-κB pathway and regulate downstream cancer-associated signals [132].

Diabetes is also a risk factor for PC. In diabetic patients, the ratio of *Firmicutes* to *Bacteroidetes* species is relatively high, affecting metabolism of carbohydrates and production of SCFAs [133]. Perry et al. demonstrated that increased levels of acetate in the blood led to insulin resistance [134], while Devaraj et al. showed that decreased levels of butyrate in the intestine promoted low-level inflammation and caused insulin resistance [135]. Decreased levels of butyrate also impaired epithelial tight junctions in the intestinal mucosa and promoted entry of bacterial endotoxins into the blood [136]. This mechanism could link diabetes and PC through endotoxemia. This effect of butyrate level was similar in individuals of different races, and metformin was able to adjust the level of butyrate effectively [137]. However, more experimental evidence is needed to confirm connections between the microbiome, diabetes and PC.

Currently, the relationship between metabolic diseases and PC has been extensively studied. However, the relationships between microbiomes and metabolic diseases are not currently sufficient to draw firm conclusions. Promisingly, we still emphasize the importance of metabolic disorders associated with microbial diversity and microbial metabolites, which are worth further exploration.

## Potential clinical application of microbiomes

### Microbiomes as diagnostic markers

Despite many studies suggesting an association between oral microbial dysbiosis and PC, no convincing evidence has indicated whether oral microbial dysbiosis is causally related to or merely an effect of early PC [138]. However, further studies of bacterial markers of periodontal disease such as *P. gingivalis* and changes in microbial diversity may suggest non-invasive screening biomarkers for PC. Recent developments suggest that salivary RNA markers can be used to identify oral bacteria by high-throughput sequencing of bacterial small subunit ribosomal RNA (16S rRNA) genes [7, 139, 140]. Therefore, saliva testing, a non-invasive test of oral biomarkers, may become a convenient strategy to screen for PC in the future. However, the existing results must be confirmed in larger multicenter prospective studies [140].

In addition, other body fluids may contain diagnostic markers of the microbiome relevant to other cancers. For example, feces can be used as a biomarker for colorectal cancer while urine may contain biomarkers of bladder cancer [141, 142]. Therefore, other body fluids such as feces, blood and pancreatic juice may also provide diagnostic markers for PC. All of these biomarkers require more study to demonstrate their potential value.

### Microbiomes as therapeutic targets

In *Fusobacterium*-associated colorectal cancer, metronidazole treatment could reduce not only the *Fusobacterium* load, but also cancer cell proliferation and patient-derived xenograft tumor growth [143]. Similarly, if there are a variety of microbes located in or associated with PC, these microbes could also become future therapeutic targets for PC. In this part, we will discuss the role of microbiomes in gemcitabine therapy, PD-1 targeted therapy, and antibiotics therapy, aiming to emphasize that some microbiomes can be seen as therapeutic targets in PC.

#### Microbiomes and gemcitabine chemoresistance

Chemotherapy is still the first-line treatment for PC of all stages, but the treatment effect differs widely in individual patients [144]. Recent studies revealed that the microbiome played an important role in determining the efficacy and side effects of chemotherapy [145, 146]. Chemotherapy could also affect the microbiome through multiple mechanisms.

Gemcitabine (2′,2′-difluorodeoxycytidine) is a representative chemotherapy drug that is widely used for treatment of various cancers including PC. However, bacteria can metabolize gemcitabine to 2′,2′-difluorodeoxyuridine, an inactive form [144], using cytidine deaminase (CDD) [147, 148]. Using deep sequencing of bacterial 16S rDNA, Geller et al. demonstrated that most of the microbes associated with pancreatic tumors were *γ-proteobacteria*, including *Enterobacter* and *Pseudomonas* species [144]. These microbes can produce CDD, leading to degradation of and resistance to gemcitabine [144]. In addition to CDD, the pyrimidine nucleoside phosphorylase (PyNP) produced by mycoplasmas also has a detrimental effect on the therapeutic efficacy of chemotherapeutic drugs by indirectly potentiating deamination of these drugs [149]. The natural pyrimidine nucleosides uridine, 2′-deoxyuridine and thymidine, which can inhibit deamination of gemcitabine, were removed by PyNP [149]. Moreover, in other cancers, certain microbes could also decrease the effect of gemcitabine. For instance, in laboratory culture, *Mycoplasma hyorhinis* contamination led to gemcitabine resistance [150, 151]. In addition, Panos et al. found that gemcitabine incubated with *E. coli* supernatants became less active [152]. Thus, the combination of antibiotics and gemcitabine may represent a new strategy to increase chemosensitivity in PC patients.

However, this does not mean the use of antibiotics is without challenges. In lymphoma, colon carcinoma and melanoma, Iida et al. showed that antibiotic-treated or germ-free mice engrafted with tumors failed to respond to CpG-oligonucleotide immunotherapy and platinum chemotherapy. Moreover, antibiotic-treated mice showed downregulation of genes related to antigen presentation and adaptive immune responses but upregulation of genes related to cancer [97]. Therefore, whether antibiotics can be used in cancer combination treatment regimens, and which antibiotics should be used, requires further study.

In addition to the ability of microbes to affect the activity of gemcitabine, the drug can also perturb the microbiome [153]. Chemotherapy is harmful to the gastrointestinal mucosa, where it may have direct cytotoxic effects on cells or produce changes in the microbiomes of the gut [154, 155]. *Firmicutes* and *Bacteroidetes* species, two dominant phyla of gut microbiomes in the normal intestine, were replaced by *Proteobacteria* and *Verrucomicrobia* in gemcitabine-treated mice, leading to gut inflammation and promoting the development of PC [5, 156–159]. Another study showed that treatment with gemcitabine can promoted infection by *Clostridium difficile*, which was undetectable in mice that were not treated with gemcitabine [5]. In addition to the microbiome itself, some studies also found that gemcitabine produces significant changes in the metabolomic profiles associated with specific microbes [160–163]. For example, Panebianco et al. found that inosine levels were significantly reduced in mice treated with gemcitabine; the mice also developed jaundice and had increased hypoxanthine levels [5]. Inosine is a natural metabolite of adenosine with anti-inflammatory and immunosuppressive functions, which has protective effects against LPS-induced inflammation [163, 164]. Therefore, destruction of the microbiome can occur during gemcitabine therapy, leading to a vicious cycle that accelerates tumor progression.

Although some progress has been made in this area, microbe-host-drug interactions are still not fully understood. Biological complexity remains a huge obstacle to precision treatment [165]. More research is needed to understand the role of the microbiome in chemotherapy resistance in PC, which has the potential to improve its poor prognosis.

#### The microbiome and PD-1-targeted therapies

Immunotherapy is effective against many malignant tumors. Immune checkpoint inhibitors can upregulate T cell responses by suppressing the T cell inhibitory receptors or their ligands on tumor cells [166]. Monoclonal antibodies targeting programmed death protein 1 (PD-1) are widely used and highly effective in melanoma, non–small cell lung cancer and renal cell carcinoma [102–104, 167, 168]. Interestingly, resistance to anti-PD-1 therapy has been observed and microbiomes may have a non-negligible effect in this process [167–169]. In non–small cell lung cancer and renal cell carcinoma, Routy et al. reported the antibiotic treatment significantly inhibited the efficacy of an anti-PD-1 monoclonal antibody [102]. They used quantitative metagenomics to explore the composition of the gut microbiomes and found that patients with *Akkermansia muciniphila* had better prognoses. Their results suggested that T helper cell 1 and cytotoxic T lymphocyte responses were positively associated with the presence of *A. muciniphila*. However, Pushalkar et al. reported opposite results regarding the effect of antibiotics in PC [4]. Their results revealed that antibiotics can enhance the anti-PD-1 effect of antibodies and enhance the activation of intratumoral CD4^+^ and CD8^+^ T cells via upregulation of PD-1 in T cells. They suggested that antibiotic therapy can be combined with checkpoint-directed immunotherapy, potentially representing a new strategy for treating patients with PC [4, 102].

In summary, these studies indicated that different microbiomes may play different roles in PC tumor microenvironments. Future studies should focus on specific categories of microbes to provide a theoretical basis for combining antibiotics with PD-1 therapy.

#### Microbiomes and antibiotics

As mentioned above, antibiotics may increase tumor sensitivity to drugs such as anti-PD-1 antibodies and gemcitabine. However, excessive exposure to antibiotics may cause dysbacteriosis and promote tumorigenesis. For instance, male patients who used tetracycline for 4 or more years had a significantly higher risk of prostate cancer. Moreover, increased risk was observed for all antibiotic classes in studies as well as in a subgroup analysis of patients who died from breast cancer [170, 171]. According to a population-based nested case-control study including 125,441 cases and 490,510 matched controls, use of penicillin was associated with elevated risk of PC [172]. The risk increased with the number of antibiotic courses but it then diminished over time [172]. By contrast, macrolides, cephalosporins, tetracyclines, antivirals, and antifungals were not associated with increased risk of PC [172]. Moreover, antibiotic-treated mice showed downregulation of genes related to antigen presentation and adaptive immune responses but upregulation of genes related to cancer [97].

Clearly, the use of antibiotics in patients with PC represents a major challenge. Whether the use of antibiotics can potentiate other treatments or promote tumor development may depend on the composition and proportion of microbiomes.

## Conclusions

PC carries a poor prognosis. Our understanding of PC has gradually advanced, and there is now some evidence that occurrence, development and therapy of PC are all related to the microbiome in vivo. The study of microbes in the pancreatic tumor microenvironment may also have potential significance for treatment of PC. The use of probiotics/antibiotics may be combined with traditional treatments such as surgery, radiotherapy and chemotherapy, as well as emerging targeted therapies and immunotherapies, to yield novel treatment options. More study is required to understand the complex relationships between the microbiome and PC.

## Data Availability

Not applicable.

## Abbreviations

AHL-12: N-acetyl-dodecanoyl homoserine
AP-1: Activator protein 1
Cag-A: Cytotoxin-associated gene-A
CDD: Cytidine deaminase
CI: Confidence interval
EPIC: European prospective investigation into cancer and nutrition
ERK1/2: Extracellular regulated protein kinases
GFR: growth factor receptors
HOMIM: Human oral microbiological identification microarrays
IL: interleukin
KC mice: K-rasLSL.G12D; Pdx1-Cre mice
KPC mice: K-rasLSL.G12D; p53R172H/+; Pdx1-Cre mice
KRAS: Kirsten rat sarcoma viral oncogene
LPS: Lipopolysaccharide
MAPK: Mitogen-activated protein kinase
mTOR: Mammalian target of rapamycin
NF-κB: Nuclear factor kappa B
NOD/SCID: Non-obese diabetic/severe combined immunodeficient
PC: Pancreatic cancer
PCN: pancreatic cystic neoplasm
PD-1: Programmed death-1
PD-L1: Programmed death-ligand 1
PyNP: Pyrimidine nucleoside phosphorylase
SCFA: short-chain fatty acids
STAT3: Signal transducers and activators of transcription 3
TbR: transforming growth factor-β receptors
TGF-β: transforming growth factor-β
Th1/2: Helper T cell 1/2
TLRs: Toll-like receptors
TNFR: tumor necrosis factor receptor
TNF-α: tumor necrosis factor-α
Wnt pathway: Wingless / Integrated pathway
